# Association between role stress and job burnout among clinical research nurses: a cross-sectional study in China

**DOI:** 10.3389/fpubh.2026.1957438

**Published:** 2026-09-23

**Authors:** Tengqiong Xiong, Lin Zheng, Tongyang You, Jian Jin, Lina Zhao, Ruilin Ding

**Affiliations:** 1 Department of Clinical Research Management, Affiliated Hospital of Southwest Medical University, Luzhou, Sichuan, China; 2 School of Nursing, Southwest Medical University, Luzhou, Sichuan, China; 3 School of Humanities and Management Science, Southwest Medical University, Luzhou, Sichuan, China; 4 Department of Emergency Medicine, Affiliated Hospital of Southwest Medical University, Luzhou, Sichuan, China

**Keywords:** clinical research nurse, job burnout, role ambiguity, role overload, role stress

## Abstract

**Objective:**

This study aimed to investigate the levels and correlates of role stress and job burnout among clinical research nurses (CRNs) in tertiary hospitals in Sichuan Province, China, and to explore the relationship between these two constructs.

**Methods:**

A cross-sectional survey was conducted from February to April 2026 in three Grade A tertiary hospitals in Sichuan Province, China. A convenience sample of 178 CRNs completed the general information questionnaire, the Role Stress Scale, and the Maslach Burnout Inventory-General Survey (MBI-GS). A Pearson's correlation was used to determine the relationship between role stress and job burnout. A linear regression analysis explored the association between role stress and job burnout.

**Results:**

The mean score rates for role stress and burnout among CRNs were 73.40% ± 15.50% and 65.96% ± 15.41%, respectively. Role conflict scored highest among role stress dimensions (75.51%), while emotional exhaustion was the most prominent burnout dimension (82.3%). Role stress was positively correlated with burnout. Multiple regression showed that role ambiguity and role overload were independently associated with job burnout.

**Conclusion:**

Our findings indicate that CRNs in tertiary hospitals in Sichuan Province experience elevated levels of both role stress and burnout. Role ambiguity and role overload were independently associated with burnout for CRNs. These results highlight the need for organizational interventions that clarify role expectations, provide systematic training, and establish clear career pathways for CRNs.

## 1 Introduction

Clinical research nurses (CRNs) are a specialized nursing group whose primary function is to support and facilitate the conduct of clinical trials (1, 2). Registered nurses in general are expected to deliver person-centered care, and CRNs, given their direct and frequent interactions with trial participants, are inevitably drawn into clinical support activities—ranging from fielding patient queries to providing hands-on nursing interventions. This convergence of research coordination and clinical practice is widely referred to as the “dual role” inherent to CRN functioning (3). In the Chinese hospital context, CRNs are operationally defined as registered nurses who hold a valid nursing license and are engaged, on either a full-time or part-time basis, in the coordination and support of clinical trial activities, including participant recruitment, protocol implementation, and data collection, irrespective of whether their substantive post is in the clinical research department or a clinical ward.

While the CRN role has gained increasing recognition internationally over recent decades, its development in China remains in its early stages and is beset with context-specific difficulties. The country has witnessed a marked rise in clinical trial activity (4), yet the CRN workforce continues to be composed predominantly of part-time staff, with limited access to structured training and no clearly defined scope of practice. These shortcomings collectively contribute to the high levels of stress experienced by CRNs (3, 5). Consequently, the accrual of multiple role stressors predisposes this group to job burnout.

Job burnout is understood as a prolonged psychological and physiological reaction to chronic emotional and interpersonal stressors inherent in the workplace (6). Its hallmark features encompass profound fatigue, growing cynicism toward professional duties, and a progressive withdrawal from social engagement at work (6, 7). From the perspective of the Job Demands-Resources model, high job demands combined with insufficient resources constitute the core pathway to burnout (8, 9). In the present study, the three role stress dimensions (role ambiguity, role conflict, and role overload) are conceptualized as job demands, whereas job resources were not directly measured; the model therefore serves as an interpretive framework for the observed associations rather than as a formally tested mechanism.

Previous studies among healthcare workers have confirmed a significant association between role stress and burnout (10, 11). For clinical nurses, certain occupational factors, including heavy workloads, understaffing, extended shifts, and limited autonomy, have been demonstrated to be consistently linked to elevated burnout levels. Although burnout among CRNs has been examined in previous studies (1), few have specifically examined role stress among CRNs or how its distinct dimensions (role ambiguity, role conflict, and role overload) relate to burnout, and the implications of their unique dual-role configuration remain largely unexplored.

Therefore, this study aimed to describe the current levels of role stress and job burnout among CRNs, examine their interrelationship, and identify factors associated with these outcomes, so as to inform evidence-based strategies for promoting CRN occupational health and workforce stability.

## 2 Methods

### 2.1 Study design and participants

This was a cross-sectional questionnaire survey conducted in three Grade A tertiary hospitals in Sichuan Province from February to April 2026. Eligible CRNs, including both those employed full-time and those working part-time, were enrolled through convenience sampling from the clinical research departments and relevant clinical wards of the three participating hospitals. All three participating institutions are major regional clinical trial sites in Sichuan Province, and uniquely feature both full-time and part-time CRNs, which aligns with the dual employment models of CRNs in current Chinese clinical practice. Due to resource accessibility constraints, we prioritized institutions with established prior research collaboration with our team and convenient on-site coordination capacity, leading to the final selection of these three Grade A tertiary general hospitals. We verified the eligible CRNs population at each participating hospital via the official nursing workforce registry maintained by each institution's nursing department: 173 CRNs were eligible at Hospital 1, 434 at Hospital 2, and 146 at Hospital 3, for a total of 753 eligible CRNs across the three sites. Considering the high routine clinical workload of CRNs, we only invited those who were on duty on the survey distribution day, free from urgent clinical tasks, and able to participate voluntarily; CRNs on leave, rotation, or external training were not approached for recruitment. Specifically, 50 invitations were distributed to eligible CRNs at Hospital 1, 120 at Hospital 2, and 30 at Hospital 3, totaling 200 invited participants.

Eligible participants met the following inclusion criteria: (a) held a valid registered nurse license; (b) had been engaged in CRN-related work for at least 3 months; and (c) provided written informed consent to participate voluntarily. Nurses were excluded if they were undergoing orientation, internship, or rotation training, or had been absent from work for an extended period prior to the survey.

### 2.2 Instruments

#### 2.2.1 General information questionnaire

A general information questionnaire, developed by the research team based on a literature review, was used to gather data on participants' demographic and work-related characteristics. This questionnaire was designed solely for descriptive purposes to characterize the sample and identify potential covariates; it is not a psychometric scale and therefore did not undergo formal validation. The items were selected based on prior nursing workforce studies and the specific contextual features of CRN practice in China. The items covered gender, age, education, years of nursing experience, years as a CRN, professional title, employment type, weekly working hours, number of trials currently managed, and whether the participant had received systematic standardized training in clinical research.

#### 2.2.2 Role stress scale

For the assessment of role stress, we employed a 13-item Chinese version of the Role Stress Scale and Role Load Scale, originally developed by Li and colleagues, which was adapted to capture three distinct dimensions: role ambiguity (3 items), role conflict (5 items), and role overload (5 items) (13). The Cronbach's α coefficients for the three dimensions were 0.927, 0.718, and 0.755, respectively; the KMO was 0.865 and Bartlett's test *P* < 0.001. Each item is rated on a 5-point Likert scale (1 = strongly disagree to 5 = strongly agree), and dimension and total scores were calculated by summing the corresponding items. Possible score ranges are 3–15 for role conflict, 5–25 for role ambiguity, 5–25 for role overload, and 13–65 for the total scale, with higher scores indicating higher role stress. This scale has been widely used among Chinese nurses and has shown good validity and reliability (13–15). In this study, the Cronbach's α was 0.781 for the total scale, and 0.768, 0.832, and 0.818 for the three dimensions respectively. Confirmatory factor analysis (CFA) supported the three-dimensional structure: χ^2^/df = 2.97, RMSEA = 0.071, CFI = 0.938, TLI = 0.921, SRMR = 0.049, with composite reliability (CR) of 0.82–0.84 and average variance extracted (AVE) of 0.50–0.60, confirming adequate construct validity in the CRN population.

#### 2.2.3 Maslach burnout inventory-general survey (MBI-GS)

Burnout was assessed using the Maslach Burnout Inventory-General Survey (MBI-GS), a well-validated tool for assessing the core components of burnout across diverse occupational groups (16). The Chinese version, adapted by Li and colleagues, preserves the original three-dimensional framework—emotional exhaustion (5 items, 0–30 total), depersonalization (4 items, 0–24 total), and personal accomplishment (6 items, 0–36 total). Each item consists of a 7-point Likert scale ranging from 0 (“never”) to 6 (“every day”). For scoring, the personal accomplishment dimension was reverse-coded by subtracting the raw score from the maximum possible score to create a “diminished personal accomplishment” score, so that higher scores on all three dimensions indicate greater burnout. The total burnout score was then calculated as the sum of the three dimension scores (emotional exhaustion + depersonalization + reverse-coded personal accomplishment), with higher total scores reflecting greater overall burnout. Psychometric evaluation by the adapting authors reported Cronbach's α values for the subscales ranging from 0.938 to 0.954, a KMO of 0.853, and a significant Bartlett's test (*P* < 0.001), demonstrating sound psychometric credentials in Chinese nursing and medical populations (17). In this study, the Cronbach's α was 0.878 for the total scale, and 0.868, 0.897, and 0.787 for the three dimensions respectively. CFA supported the three-dimensional structure: χ^2^/df = 2.82, RMSEA = 0.065, CFI = 0.947, TLI = 0.932, SRMR = 0.042, with CR of 0.78–0.87 and AVE of 0.39–0.58, confirming adequate construct validity.

### 2.3 Data collection

Ethical approval was obtained from the Institutional Ethics Committees of the Affiliated Hospital of Southwest Medical University (Luzhou, China; approval number: KY2025745). The approved protocol specified recruitment and questionnaire-based data collection at three tertiary Grade A hospitals in Sichuan province. Each participating hospital reviewed the approved protocol and granted written site permission before recruitment. According to the institutional policies of the participating hospitals, separate local ethics approval was not required because the study was approved by the lead ethics committee and involved minimal-risk questionnaire-based data collection. Written informed consent was obtained from all participants prior to participation.

The participants completed the questionnaires via a QR code generated on the network platform, taking approximately 8–10 min. To ensure data quality, all items were mandatory; duplicate submissions were prevented by restricting one submission per device without collecting any personally identifying information beyond that needed for the survey; and questionnaires with a completion time of less than 3 mins (considered too short to have been completed thoughtfully) or with identical responses were excluded. Before filling out the questionnaire, all participants received a standardized instruction session that clearly explained the quality criteria and exclusion rules. Because all items were mandatory, no item-level missing data were expected. Of the invited CRNs, hospital 1 returned 43 questionnaires, 1 of which was excluded as invalid, yielding 42 valid responses (response rate: 86.00%, effective rate: 97.67%). Hospital 2 returned 108 questionnaires, 2 of which were excluded as invalid, yielding 106 valid responses (response rate: 90.00%, effective rate: 98.15%). Hospital 3 returned all 30 distributed questionnaires with no invalid submissions, achieving a 100.0% response and effective rate. Across the full sample, 178 valid questionnaires were obtained from 181 returned submissions, corresponding to an overall response rate of 90.50% and an effective rate of 98.34%.

### 2.4 Sample size estimation

An *a priori* power analysis was conducted using G^*^Power 3.1 to determine the required sample size for the multiple linear regression with burnout as the dependent variable (12). Based on Cohen's (1988) guidelines, the following parameters were specified: significance level (α) = 0.05 (two-tailed), desired statistical power = 0.80, number of predictors (k) = 14 (11 demographic and work-related variables plus 3 role stress dimensions: role conflict, role ambiguity, and role overload; the total role stress score was not entered as a separate predictor due to multicollinearity with its constituent dimensions, and burnout dimensions were correctly excluded as they served as the dependent variable), and effect size (f^2^) = 0.15 (conservative medium effect). The minimum required sample size was calculated as *N* = 166. This estimate is intentionally conservative. Our final analytic cohort consisted of 178 valid responses, exceeding the minimum requirement of 166 and providing adequate power (≥ 80%) to detect medium-to-large effects in the planned 14-predictor linear regression model.

### 2.5 Statistical analysis

Data were analyzed using SPSS 27.0. Categorical variables were presented as frequencies and percentages. Normality was assessed using the Kolmogorov-Smirnov test together with visual inspection of Q-Q plots. Continuous variables following a normal distribution were expressed as mean ± standard deviation (SD); otherwise, median and interquartile range were used. Score rate = (actual score / total possible score) × 100%. Levels of burnout and role stress were described using the score rate. Pearson correlation was used to examine relationships between role stress and burnout dimensions.

Univariate comparisons were performed using independent *t*-tests or one-way ANOVA. Candidate variables were selected for the multiple linear regression on the basis of prior evidence and the study's conceptual framework; variables with *P* < 0.05 in univariate analysis were entered into the model, and a backward stepwise procedure was applied to obtain a parsimonious model. A sensitivity analysis was also performed by forcing all prespecified covariates into the model simultaneously, regardless of their univariate significance, to assess the robustness of the findings.

The assumptions of multiple linear regression were assessed. Normality of residuals was examined using the Kolmogorov-Smirnov test. Independence of residuals was assessed using the Durbin-Watson statistic. Influential observations were identified by Cook's distance. Multicollinearity was evaluated using variance inflation factors (VIF) (18), with a threshold of 5 as the upper acceptable limit.

Given the number of univariate and correlational comparisons, the risk of Type I error was considered. Univariate and correlational analyses were treated as exploratory and hypothesis-generating; therefore, no adjustment for multiple testing (e.g., Bonferroni correction) was applied, as such adjustment would increase the risk of Type II error and potentially obscure meaningful associations. By contrast, the multiple linear regression was the prespecified primary analysis, with burnout as the dependent variable, and significance was set at α = 0.05 (two-tailed).

## 3 Results

### 3.1 Characteristics of the participants

A total of 178 CRNs participated in the study. The sample comprised 11 males (6.18%) and 167 females (93.82%), with ages ranging from 24 to 46 years. 77 participants (43.26%) were employed as full-time CRNs, while the remaining 101 (56.74%) worked in part-time capacities. The majority held a bachelor's degree (51.69%) or a master's degree (33.71%), together representing 85.40% of the cohort. All participants completed the questionnaire independently. Detailed demographic characteristics are summarized in Table 1.

**Table 1:** Sample characteristics and differences in job burnout (*n* = 178).

Factors	Frequency (%)	Job burnout scores	Statistic	*P* value
Gender				
Male	11 (6.18)	63.55 ± 11.29	*t* = 1.034	0.303
Female	167 (93.82)	59.08 ± 14.01		
Age (years)				
24–30	69 (38.76)	58.97 ± 14.39	F = 3.029	0.031
31–35	52 (29.21)	62.33 ± 13.73		
36–40	41 (23.03)	57.91 ± 12.43		
41–46	16 (8.99)	40.00 ± 6.08		
Educational level				
Diploma	22 (12.36)	61.91 ± 14.63	F = 0.643	0.589
Bachelor's degree	92 (51.69)	58.59 ± 14.95		
Master's degree	60 (33.71)	60.02 ± 11.92		
Doctoral degree	4 (2.25)	53.25 ± 12.47		
Work experience (years)				
1–3	45 (25.28)	59.24 ± 12.88	F = 2.831	0.026
4–5	55 (30.90)	62.60 ± 13.53		
6–8	35 (19.66)	59.49 ± 13.86		
9–10	12 (6.74)	62.08 ± 14.16		
>10	31 (17.42)	52.58 ± 14.19		
Years as a CRN (years)				
< 1	26 (14.61)	57.27 ± 12.98	F = 0.997	0.410
1–2	54 (30.34)	60.72 ± 13.67		
3–5	51 (28.65)	60.51 ± 14.70		
6–10	37 (20.79)	59.14 ± 13.89		
>10	10 (5.62)	52.40 ± 12.55		
Professional title				
Primary nurse	75 (42.13)	63.04 ± 12.99	F = 3.262	0.013
Nurse practitioner	57 (32.02)	54.67 ± 15.06		
Supervisor nurse	29 (16.29)	58.00 ± 11.87		
Co–chief nurse	8 (4.49)	60.50 ± 15.18		
Chief nurse	9 (5.06)	61.78 ± 10.71		
Employment type				
Bianzhi^*^	83 (46.63)	59.83 ± 12.68	F = 2.442	0.090
Contract–based employment	92 (51.69)	59.50 ± 14.78		
Others	3 (1.69)	42.00 ± 6.56		
Weekly working hours				
≤ 20	14 (7.87)	55.93 ± 10.17	F = 0.953	0.416
21–39	50 (28.09)	59.94 ± 13.98		
40–49	59 (33.15)	57.80 ± 15.80		
≥ 50	55 (30.90)	61.38 ± 12.25		
Number of trials currently managed				
1–2	80 (44.94)	60.44 ± 12.55	F = 0.314	0.815
3–4	57 (32.02)	58.77 ± 13.57		
5–6	23 (12.92)	58.30 ± 17.03		
>6	18 (10.11)	57.78 ± 16.69		
Systematic standardized training of clinical research				
Yes	122 (68.54)	58.08 ± 13.31	*t* = −1.826	0.070
No	56 (31.46)	62.14 ± 14.75		
Employment status				
Full–time job	77 (43.26)	59.36 ± 14.01	*t* = 0.003	0.997
Part–time job	101 (56.74)	59.36 ± 13.83		

^*^Bianzhi, a state-guaranteed permanent position with lifetime employment and comprehensive benefits; Data were presented as mean ± SD.

### 3.2 Univariate analysis of the different general information on job burnout of CRNs

The mean total burnout score among participants was 59.36 ± 13.87, corresponding to a score rate of 65.96% ± 15.41%. Dimension-wise scores were as follows (Table 2): emotional exhaustion, 24.68 ± 8.40 (score rate: 82.27% ± 28.00%); depersonalization, 18.69 ± 7.37 (77.88% ± 30.71%); and diminished personal accomplishment, 15.99 ± 8.42 (44.41% ± 23.39%). This pattern indicates that the burnout experienced by CRNs was driven primarily by emotional depletion and interpersonal detachment rather than by a loss of professional confidence.

**Table 2:** CRN job burnout dimension scores and score rate.

Dimensions	Total possible score	Actual score	Score rate (%)
Emotional exhaustion	30	24.68 ± 8.40	82.27 ± 28.00
Depersonalization	24	18.69 ± 7.37	77.88 ± 30.71
Diminished personal accomplishment	36	15.99 ± 8.42	44.41 ± 23.39
Job burnout	90	59.36 ± 13.87	65.96 ± 15.41

Univariate analysis showed that age, work experience, and professional title emerged as significant correlates of burnout (Table 1, Supplementary Table S1). Specifically, CRNs aged 41–46 years exhibited lower depersonalization and total burnout scores. Longer nursing experience was significantly associated with lower depersonalization and total burnout. In terms of professional title, nurse practitioners scored significantly lower on emotional exhaustion, depersonalization, and total burnout. In contrast, total burnout scores did not differ significantly across gender, educational level, years of CRN experience, employment type, weekly working hours, number of trials managed, receipt of systematic standardized training, or full-time versus part-time status, although some individual burnout dimensions (notably depersonalization) did differ significantly for certain variables, as described below. Regarding working hours, those working ≤ 20 h per week had lower depersonalization scores. Additionally, participants without systematic training of clinical research reported higher depersonalization (Supplementary Table S1).

### 3.3 Analysis of role stress in CRNs

The mean total role stress score among participants was 47.71 ± 10.08, yielding a score rate of 73.40% ± 15.50%. Detailed scores for each dimension of role stress are shown in Table 3.

**Table 3:** CRN role stress dimension scores and score rate.

Dimensions	Total possible score	Actual score	Score rate (%)
Role ambiguity	25	17.82 ± 6.11	71.26 ± 24.44
Role conflict	15	11.33 ± 3.00	75.51 ± 20.03
Role overload	25	18.58 ± 4.44	74.30 ± 17.74
Role stress	65	47.71 ± 10.08	73.40 ± 15.50

As summarized in Supplementary Table S2, the analysis revealed that total role stress scores, as well as scores on specific dimensions, differed significantly according to age, educational level, nursing experience, professional title, weekly working hours, employment type, and whether participants had received systematic standardized training. In contrast, gender, years of CRN experience, and number of trials managed showed no significant associations with either the total score or any subdimension scores (all *P* > 0.05).

### 3.4 Correlation between role stress and job burnout

Pearson correlation analysis (Table 4) revealed that total role stress was positively associated with total burnout (*r* = 0.370, *P* < 0.001), emotional exhaustion (*r* = 0.327, *P* < 0.001), and depersonalization (*r* = 0.543, *P* < 0.001), whereas a negative association was observed with diminished personal accomplishment (*r* = −0.192, *P* = 0.010).

**Table 4:** Correlations between role stress and job burnout (*n* = 178, *r* value).

Variables	Role ambiguity	Role conflict	Role overload	Total score of role stress	Emotional exhaustion	Depersonalization	Diminished personal accomplishment	Total score of job burnout
Role ambiguity	1	0.153^*^	0.294^**^	0.782^**^	0.306^**^	0.602^**^	−0.206^*^	0.380^**^
Role conflict		1	0.522^**^	0.621^**^	0.087	0.166^*^	−0.103	0.078
Role overload			1	0.774^**^	0.263^**^	0.290^**^	−0.081	0.264^**^
Total score of role stress				1	0.327^**^	0.543^**^	−0.192^*^	0.370^**^
Emotional exhaustion					1	0.377^**^	−0.227^*^	0.668^**^
Depersonalization						1	−0.144	0.672^**^
Diminished personal accomplishment							1	0.393^**^
Total score of job burnout								1

^*^P < 0.01, ^**^P < 0.001.

When examined by individual role stress dimensions, role ambiguity showed positive correlations with emotional exhaustion (*r* = 0.306, *P* < 0.01), depersonalization (*r* = 0.602, *P* < 0.001), and total burnout (*r* = 0.380, *P* < 0.001), but was inversely related to personal accomplishment (*r* = −0.206, *P* < 0.01). Role conflict was found to correlate significantly only with depersonalization (*r* = 0.166, *P* < 0.01). Role overload was positively correlated with emotional exhaustion (*r* = 0.263, *P* < 0.001), depersonalization (*r* = 0.290, *P* < 0.001), and total burnout (*r* = 0.264, *P* < 0.001).

With respect to within-scale intercorrelations, the three role stress dimensions were all positively intercorrelated. Among burnout subscales, emotional exhaustion was positively correlated with depersonalization (*r* = 0.377, *P* < 0.001) and negatively with diminished personal accomplishment (*r* = −0.227, *P* < 0.001).

### 3.5 Multiple linear regression for job burnout

A backward stepwise multiple linear regression was performed with total burnout score as the dependent variable and age, nursing experience, professional title, role ambiguity, role conflict, and role overload as independent variables. Variables with *P* > 0.05 were sequentially removed. Age, professional title, nursing experience, and role conflict were eliminated in this order. The final model retained role ambiguity and role overload. The model was statistically significant (*F* = 17.905, *P* < 0.001, adjusted *R*^2^ = 0.260) and accounted for 26.0% of the variance in burnout scores. Role ambiguity [B = 0.751, *P* < 0.001; 95% CI (0.428–1.074)] and role overload [B = 0.522, *P* = 0.022; 95% CI (0.077-0.967)] were independently associated with burnout (Table 5). All regression assumptions were satisfied. The Durbin-Watson statistic was 2.019, indicating independent residuals. The Kolmogorov-Smirnov test confirmed normality of residuals (D = 0.060, *P* = 0.200). The maximum Cook's distance was 0.098, below the threshold of 1, indicating no influential outliers. VIF values for role ambiguity and role overload were 1.074 and 1.095, respectively, well below the threshold of 5, confirming no multicollinearity.

**Table 5:** Multiple linear regression for job burnout total score.

Factors	Unstandardised B (95% confidence intervals)	Unstandardised SE	β	*t*	*P* value	VIF
(Constant)	36.287 (27.521, 45.052)	4.441	–	8.170	< 0.001	–
Role ambiguity	0.751 (0.428, 1.074)	0.164	0.331	4.592	< 0.001	1.074
Role overload	0.522 (0.077, 0.967)	0.225	0.167	2.317	0.022	1.095

VIF, Variance Inflation Factor

In a sensitivity analysis, all prespecified covariates were forced into the model simultaneously; the effect sizes and significance of role ambiguity and role overload remained stable (Supplementary Table S3).

## 4 Discussion

This study investigated the levels of role stress and job burnout among CRNs in three tertiary hospitals in Sichuan Province, and examined the relationship between these two constructs. The findings contribute empirical evidence on CRN occupational wellbeing—an area that has received limited attention in China—and offer practical implications for workforce management in clinical research settings.

CRNs in our sample reported an overall burnout score rate of 65.96% ± 15.41%. This figure is somewhat higher than that reported in studies of general clinical nurses in China, where total burnout score rates typically range from 40% to 60% (19–21). The elevated burnout level among CRNs is perhaps unsurprising given the dual demands of their role. Unlike general nurses, who primarily attend to patient care, CRNs must simultaneously navigate protocol-driven research requirements, regulatory compliance, and the unpredictable nature of clinical trials—all while maintaining patient-centered care.

In terms of dimensional patterns, emotional exhaustion emerged as the most pronounced dimension, followed by depersonalization, while diminished personal accomplishment scored substantially lower. This pattern is consistent with previous studies, in which emotional exhaustion is consistently identified as the primary manifestation of burnout, reflecting the emotionally demanding nature of patient-facing roles (22). The high emotional exhaustion score among CRNs suggests that the sustained emotional labor involved in interacting with trial participants—many of whom may be seriously ill or facing uncertain outcomes—combined with the pressure of maintaining protocol fidelity, exacts a considerable toll on CRNs' emotional resources. The comparatively low score on diminished personal accomplishment is noteworthy. One possible explanation lies in the nature of clinical research work. CRNs are directly involved in bringing new therapies to patients, and the tangible contributions to medical progress may provide intrinsic rewards that buffer against feelings of ineffectiveness (23, 24). Another contributing factor may be the educational composition of our sample—over 85% of participants held a bachelor's or master's degree. Higher educational attainment has been shown to be associated with stronger professional identity and more comprehensive self-perceptions, which may protect against the erosion of personal accomplishment (25). These explanations are offered as tentative hypotheses and were not directly measured in the present study.

Previous studies have consistently identified role stress as a significant contributor to job burnout (26, 27). In this sample, the total role stress score rate was 73.40% ± 15.50%, comparable to that reported in general Chinese nursing populations (13, 28), suggesting that role stress is a common occupational psychological issue across nursing specialties. Among the dimensions, role conflict scored highest, followed by role overload and role ambiguity. This pattern is consistent with the qualitative observations of Backman Lönn et al. (29), who characterized CRNs as “the hub of a wheel without real power,” underscoring the role-responsibility mismatch that underlies elevated role stress. The association between role stress and burnout was further examined using Pearson correlation analysis. At the aggregate level, total role stress correlated positively with total burnout, emotional exhaustion, and depersonalization, and negatively with diminished personal accomplishment. Notably, the three role stress dimensions exhibited distinct patterns of association with the burnout subscales. This pattern of associations is consistent with the Job Demands-Resources model, which posits that high job demands deplete emotional resources and foster cynical attitudes (9).

Univariate analysis identified age, years of nursing experience, professional title, total role stress score, role overload score, and role ambiguity score as significant correlates of job burnout. Our findings showed that CRNs aged 41–46 years and those with longer nursing experience (≥ 10 years) exhibited lower depersonalization and total burnout scores. This age- and experience-related pattern is consistent with the previous nursing literature, where older and more experienced nurses tend to report lower levels of depersonalization (30, 31). The observed pattern of burnout across professional titles, with nurse practitioners scoring significantly lower than both junior nurses and chief nurse practitioners. Mid-career professionals may possess an optimal combination of experience and energy. Junior nurses, by contrast, may lack the coping resources that develop with time and exposure, whereas chief nurse practitioners, despite their seniority, often assume heightened managerial responsibilities that impose additional emotional and cognitive demands.

With respect to role stress, role overload and role ambiguity emerged as significant correlates with job burnout. Role overload, reflecting the sheer volume of tasks that exceed available time and capacity (32), directly depletes CRNs' physical and emotional energy, which manifests as elevated burnout scores. Role ambiguity, by contrast, operates through a different mechanism. Unlike clinical nurses with well-circumscribed duties, CRNs are expected to function simultaneously as care providers and research coordinators—two domains that often operate under distinct logics, timelines, and performance metrics. When the boundaries between these roles remain unclear, CRNs are compelled to invest substantial cognitive and emotional resources in determining which set of expectations to prioritize at any given moment, a task that is inherently more demanding than simply managing a single, well-defined role (3). This “role-making” burden may be particularly taxing in the Chinese context, where the CRN role remains nascent and lacks standardized training and formal recognition. Notably, role conflict did not emerge as a significant factor in the univariate analysis. A plausible explanation is that role conflict, stemming from the inherent duality of CRN practice, may be a relatively uniform experience across the sample, with less interindividual variation than role ambiguity or role overload. Alternatively, its effect on burnout may be indirect, operating through other role stress dimensions; however, this hypothesis was not tested and requires further investigation. This interpretation is consistent with the observed correlation pattern, wherein role conflict correlated significantly only with depersonalization, whereas role ambiguity and role overload showed broader associations with multiple burnout dimensions.

After multiple linear regression, role ambiguity and role overload were independently associated with job burnout. The stronger association of role ambiguity relative to role overload with burnout is a particularly important finding. It suggests that for CRNs, uncertainty about role expectations may be more damaging to psychological wellbeing than simply having too much work—even though the latter is also a significant contributor. Age, nursing experience, and professional title, although significant in univariate analyses, were non-significant in the regression model once role stress dimensions were entered. This loss of significance after adjustment does not by itself establish mediation, and the possibility of indirect pathways through role stress perceptions should be tested formally in future work. The proportion of variance explained (26.0%) also suggests that other factors, including organizational support, perceived autonomy, supervisor relationships, and individual psychological resources, contribute to burnout, and future research should explore these additional pathways.

The present study has several limitations. First, the cross-sectional design precludes causal inferences regarding the relationships between role stress and burnout. Second, the sample was drawn from only three tertiary hospitals in a single province, which limits the generalisability of our findings to other regions in China. Third, the use of convenience sampling may have introduced selection bias. Fourth, reliance on self-report measures collected at a single time point raises the possibility of common method bias, which may have inflated the observed correlations between role stress and burnout. Although procedural precautions, such as anonymous completion, standardized instructions, and data-quality screening, were implemented, no statistical test (e.g., Harman's single-factor test) was performed to quantify the extent of common method variance. Consequently, the correlation coefficients, particularly between self-reported role stress and burnout, should be interpreted with caution, as they may overestimate the true relationships. Future research should employ multi-source data (e.g., supervisor-rated burnout or objective workload measures) or longitudinal designs to disentangle causal pathways and reduce same-source bias. Finally, the large number of univariate and correlational tests conducted may have increased the risk of Type I error. We acknowledge that some significant findings in these exploratory analyses may have arisen by chance. To mitigate this concern, we prespecified the multiple linear regression as the primary confirmatory analysis and interpreted exploratory results cautiously, emphasizing consistency across analytical approaches rather than relying on individual *P* values. Future studies with larger samples and pre-registered hypotheses are needed to confirm these findings

## 5 Conclusion

This cross-sectional study examined the levels of role stress and job burnout among CRNs in tertiary hospitals in Sichuan Province, China, and the relationship between the two constructs. Our findings indicate that CRNs experience elevated levels of both role stress and burnout. Role ambiguity and role overload were independently associated with burnout for CRNs. These results highlight the need for organizational interventions that clarify role expectations, provide systematic training, and establish clear career pathways for CRNs. Such measures are important for safeguarding the wellbeing of this specialized workforce and may, in turn, support the effective delivery of clinical research.

## Data Availability

The original contributions presented in the study are included in the article/Supplementary material, further inquiries can be directed to the corresponding author.
