# Supplementary material for: Association between role stress and job burnout among clinical research nurses: a cross-sectional study in China
Source: Front Public Health. 2026 Sep 23;14:1957438. doi: 10.3389/fpubh.2026.1957438 (PMC13642617; doi:10.3389/fpubh.2026.1957438)
Supplement: Supplementary file 1 [file Table_1.docx]

**Association between role stress and job burnout among clinical research nurses: a cross-sectional study in China**

Tengqiong Xiong^1‖^, Lin Zheng^2‖^, Tongyang You^1^, Jian Jin^3^, Lina Zhao^4,^ Ruilin Ding^1^

^1^Department of Clinical Research Management, Affiliated Hospital of Southwest Medical University, Luzhou, Sichuan 646000, P.R. China; ^2^School of Nursing, Southwest Medical University, Luzhou, Sichuan 646000, P.R. China; School of Humanities and Management Science, Southwest Medical University, Luzhou, Sichuan 646000, P.R. China; ^4^Department of Emergency Medicine, Affiliated Hospital of Southwest Medical University, Luzhou, Sichuan 646000, P.R. China

^*^Correspondence to: Ruilin Ding, Affiliated Hospital of Southwest Medical University, No 25 Taiping Street, Luzhou, Sichuan 646000, P.R. China; E-mail: [rainingdean@163.com](mailto:rainingdean@163.com);

^‖^Contributed equally

Table S1 The analysis of the job burnout scores across the sample variables

| Factors | Emotional exhaustion score | Depersonalization score | Diminished personal accomplishment score | Total score |
| --- | --- | --- | --- | --- |
| Gender |  |  |  |  |
| Male | 25.82 ± 8.05 | 21.55 ± 6.77 | 16.18 ± 10.74 | 63.55 ± 11.29 |
| Female | 24.61 ± 8.44 | 18.50 ± 7.39 | 15.98 ± 8.28 | 59.08 ± 14.01 |
| *t* | 0.463 | 1.435 | 0.078 | 1.034 |
| *P* | 0.644 | 0.178 | 0.938 | 0.303 |
| Age (years) |  |  |  |  |
| 24-30 | 24.22 ± 8.90 | 18.65 ± 7.44 | 16.10 ± 8.39 | 58.97 ± 14.38 |
| 31-35 | 25.25 ± 8.29 | 20.25 ± 6.81 | 16.83 ± 8.56 | 62.33 ± 13.73 |
| 36-40 | 25.24 ± 7.69 | 17.91 ± 7.14 | 14.76 ± 8.30 | 57.91 ± 12.43 |
| 41-46 | 18.00 ± 7.94 | 4.67 ± 1.16 | 17.33 ± 11.02 | 40.00 ± 6.08 |
| *F* | 0.855 | 4.868 | 0.524 | 3.029 |
| *P* | 0.466 | 0.003 | 0.666 | 0.031 |
| Educational level |  |  |  |  |
| Diploma | 25.91 ± 7.16 | 19.46 ± 7.21 | 16.55 ± 9.59 | 61.91 ±14.63 |
| Bachelor’s degree | 23.80 ± 9.25 | 17.57 ± 7.87 | 17.22 ± 8.83 | 58.59 ± 14.95 |
| Master’s degree | 25.73 ± 7.32 | 20.22 ± 6.40 | 14.07 ± 7.28 | 60.02 ± 11.92 |
| Doctoral degree | 22.25 ± 9.64 | 17.50 ± 8.10 | 13.50 ± 1.00 | 53.25 ± 12.47 |
| *F* | 0.915 | 1.706 | 1.871 | 0.643 |
| *P* | 0.435 | 0.167 | 0.136 | 0.589 |
| Work experience (years) |  |  |  |  |
| 1-3 | 24.96 ± 8.50 | 18.67 ± 7.40 | 15.62 ± 5.81 | 59.24 ± 12.88 |
| 4-5 | 26.15 ± 7.91 | 20.62 ± 6.21 | 15.84 ± 8.34 | 62.60 ± 13.53 |
| 6-8 | 25.14 ± 8.09 | 20.34 ± 6.81 | 14.00 ± 7.50 | 59.49 ± 13.86 |
| 9-10 | 25.33 ± 9.07 | 20.50 ± 6.45 | 16.25 ± 8.50 | 62.08 ± 14.16 |
| > 10 | 20.93 ± 8.62 | 12.74 ± 7.42 | 18.94 ± 10.22 | 52.58 ± 14.19 |
| *F* | 2.092 | 7.596 | 1.482 | 2.831 |
| *P* | 0.084 | <0.001 | 0.210 | 0.026 |
| Years as a CRN (years) |  |  |  |  |
| < 1 | 25.12 ± 7.60 | 17.96 ± 7.54 | 14.19 ± 6.28 | 57.27 ± 12.98 |
| 1-2 | 24.50 ± 8.97 | 18.96 ± 7.38 | 17.26 ± 9.13 | 60.72 ± 13.67 |
| 3-5 | 24.06 ± 9.05 | 19.67 ± 7.19 | 16.78 ± 9.01 | 60.51 ± 14.70 |
| 6-10 | 26.08 ± 7.21 | 19.03 ± 7.15 | 14.03 ± 7.70 | 59.14 ± 13.89 |
| > 10 | 22.50 ± 8.62 | 12.90 ± 7.22 | 17.00 ± 8.12 | 52.40 ± 12.55 |
| *F* | 0.513 | 1.907 | 1.263 | 0.997 |
| *P* | 0.726 | 0.111 | 0.286 | 0.410 |
| Professional title |  |  |  |  |
| Primary nurse | 26.77 ± 7.46 | 20.76 ± 6.32 | 15.51 ± 7.85 | 63.04 ± 12.99 |
| Nurse practitioner | 21.28 ± 9.40 | 15.84 ± 8.12 | 17.54 ± 9.34 | 54.67 ± 15.06 |
| Supervisor nurse | 25.00 ± 7.73 | 19.00 ± 6.75 | 14.00 ± 6.40 | 58.00 ± 11.87 |
| Co-chief nurse | 25.88 ± 6.42 | 17.13 ± 8.41 | 17.50 ± 12.81 | 60.50 ± 15.19 |
| Chief nurse | 26.67 ± 7.26 | 19.89 ± 6.88 | 15.22 ± 8.03 | 61.78 ± 10.71 |
| *F* | 3.917 | 4.027 | 1.036 | 3.262 |
| *P* | 0.005 | 0.004 | 0.390 | 0.013 |
| Employment type |  |  |  |  |
| Bianzhi* | 24.78 ± 8.04 | 19.52 ± 6.92 | 15.53 ± 8.44 | 59.83 ± 12.68 |
| Contract-based employment | 25.00 ± 8.57 | 18.13 ± 7.65 | 16.37 ± 8.51 | 59.50 ± 14.78 |
| Others | 12.00 ± 2.65 | 13.00 ± 9.54 | 17.00 ± 6.56 | 42.00 ± 6.56 |
| *F* | 3.593 | 1.696 | 0.237 | 2.442 |
| *P* | 0.030 | 0.186 | 0.789 | 0.090 |
| Weekly working hours |  |  |  |  |
| ≤ 20 | 21.79 ± 7.78 | 13.14 ± 7.25 | 21.00 ± 9.83 | 55.93 ± 10.17 |
| 21-39 | 24.38 ± 8.81 | 19.48 ± 7.60 | 16.08 ± 8.56 | 59.94 ± 13.98 |
| 40-49 | 24.27 ± 8.84 | 18.64 ± 7.53 | 14.92 ± 7.38 | 57.80 ± 15.80 |
| ≥ 50 | 26.16 ± 7.59 | 19.44 ± 6.55 | 15.78 ± 8.73 | 61.38 ± 12.25 |
| *F* | 1.206 | 3.133 | 2.020 | 0.953 |
| *P* | 0.309 | 0.027 | 0.113 | 0.416 |
| Number of trials currently managed |  |  |  |  |
| 1-2 | 23.94 ± 8.38 | 19.55 ± 7.08 | 16.95 ± 9.01 | 60.44 ±12.55 |
| 3-4 | 26.18 ± 7.68 | 18.23 ± 7.46 | 14.37 ± 6.74 | 58.77 ±13.57 |
| 5-6 | 24.35 ± 9.25 | 18.30 ± 7.48 | 15.65 ± 9.09 | 58.30 ±17.03 |
| > 6 | 23.67 ± 9.58 | 16.83 ± 8.28 | 17.28 ± 9.45 | 57.78 ±16.69 |
| *F* | 0.908 | 0.837 | 1.209 | 0.314 |
| *P* | 0.438 | 0.475 | 0.308 | 0.815 |
| Systematic standardized training of clinical research |  |  |  |  |
| Yes | 24.75 ± 8.32 | 17.67 ± 7.71 | 15.66 ± 8.17 | 58.08 ±13.31 |
| No | 24.52 ± 8.64 | 20.91 ± 6.06 | 16.71 ± 8.96 | 62.14 ±14.75 |
| *t* | 0.174 | -3.029 | -0.778 | -1.826 |
| *P* | 0.862 | 0.003 | 0.438 | 0.070 |
| Employment status |  |  |  |  |
| Full-time job | 24.56 ± 8.15 | 17.61 ± 7.56 | 17.20 ± 8.95 | 59.36 ± 14.01 |
| Part-time job | 24.77 ± 8.63 | 19.52 ± 7.15 | 15.07 ± 7.91 | 59.36 ± 13.83 |
| *t* | -0.168 | -1.704 | 1.649 | 0.003 |
| *P* | 0.867 | 0.090 | 0.101 | 0.997 |

Note: *Bianzhi, a state-guaranteed permanent position with lifetime employment and comprehensive benefits; Data were presented as mean ± SD.

Table S2 The analysis of the role stress scores across the sample variables

| Factors | Role ambiguity score | Role conflict score | Role overload score | Total score |
| --- | --- | --- | --- | --- |
| Gender |  |  |  |  |
| Male | 18.46 ± 5.32 | 10.91 ± 3.24 | 18.91 ± 3.42 | 48.27 ± 7.73 |
| Female | 17.77 ± 6.17 | 11.35 ± 3.00 | 18.55 ± 4.50 | 47.67 ± 10.23 |
| *t* | 0.358 | -0.474 | 0.263 | 0.191 |
| *P* | 0.721 | 0.655 | 0.793 | 0.848 |
| Age (years) |  |  |  |  |
| 24-30 | 18.05 ± 6.03 | 11.52 ± 2.95 | 18.92 ± 4.11 | 48.49 ± 9.25 |
| 31-35 | 18.21 ± 5.69 | 10.96 ± 3.31 | 18.40 ± 4.65 | 47.58 ± 10.16 |
| 36-40 | 17.81 ± 6.13 | 11.44 ± 2.72 | 18.35 ± 4.53 | 47.59 ± 10.69 |
| 41-46 | 5.00 ± 0.00 | 11.00 ± 4.00 | 15.67 ± 8.33 | 31.67 ± 12.22 |
| *F* | 4.802 | 0.389 | 0.649 | 2.777 |
| *P* | 0.003 | 0.761 | 0.585 | 0.043 |
| Educational level |  |  |  |  |
| Diploma | 17.82 ± 6.12 | 10.96 ± 3.51 | 19.46 ± 3.84 | 48.23 ± 9.33 |
| Bachelor’s degree | 17.01 ± 6.55 | 11.04 ± 3.01 | 17.67 ± 4.82 | 45.73 ± 10.90 |
| Master’s degree | 18.82 ± 5.42 | 11.92 ± 2.76 | 19.47 ± 3.89 | 50.20 ± 8.67 |
| Doctoral degree | 21.25 ± 2.63 | 11.00 ± 3.37 | 20.75 ± 2.06 | 53.00 ± 3.92 |
| *F* | 1.503 | 1.176 | 2.762 | 2.884 |
| *P* | 0.215 | 0.320 | 0.044 | 0.037 |
| Work experience (years) |  |  |  |  |
| 1-3 | 18.49 ± 5.57 | 11.62 ± 2.82 | 19.64 ± 3.24 | 49.76 ± 7.72 |
| 4-5 | 19.20 ± 5.14 | 11.49 ± 2.97 | 18.89 ± 4.04 | 49.58 ± 8.95 |
| 6-8 | 19.77 ± 4.85 | 11.51 ± 3.44 | 19.06 ± 2.25 | 50.34 ± 6.95 |
| 9-10 | 19.17 ± 5.02 | 12.00 ± 2.09 | 18.42 ± 4.17 | 49.58 ± 9.90 |
| > 10 | 11.65 ± 6.51 | 10.13 ± 2.96 | 15.94 ± 5.93 | 37.70 ± 12.29 |
| *F* | 12.289 | 1.588 | 3.800 | 11.469 |
| *P* | <0.001 | 0.180 | 0.005 | <0.001 |
| Years as a CRN (years) |  |  |  |  |
| < 1 | 17.42 ± 6.15 | 11.08 ± 3.10 | 18.92 ± 4.58 | 47.42 ± 10.87 |
| 1-2 | 18.82 ± 5.54 | 11.41 ± 3.17 | 18.82 ± 3.76 | 49.04 ± 8.70 |
| 3-5 | 17.12 ± 6.60 | 11.22 ± 3.09 | 18.22 ± 4.76 | 46.55 ± 11.08 |
| 6-10 | 18.35 ± 6.04 | 11.97 ± 2.47 | 18.70 ± 4.47 | 49.03 ± 9.76 |
| > 10 | 15.00 ± 6.41 | 9.70 ± 3.09 | 17.60 ± 6.10 | 42.30 ± 10.11 |
| *F* | 1.161 | 1.240 | 0.287 | 1.296 |
| *P* | 0.330 | 0.296 | 0.886 | 0.273 |
| Professional title |  |  |  |  |
| Primary nurse | 19.55 ± 4.55 | 11.76 ± 2.85 | 19.49 ± 3.29 | 50.80 ± 6.60 |
| Nurse practitioner | 15.39 ± 7.33 | 10.67 ± 3.26 | 17.26 ± 5.41 | 43.32 ± 12.36 |
| Supervisor nurse | 18.21 ± 5.97 | 11.55 ± 2.67 | 18.59 ± 4.48 | 48.35 ± 10.46 |
| Co-chief nurse | 15.63 ± 6.84 | 10.38 ± 3.20 | 18.00 ± 5.32 | 44.00 ± 11.01 |
| Chief nurse | 19.44 ± 3.84 | 12.00 ± 3.08 | 19.56 ± 3.75 | 51.00 ± 5.48 |
| *F* | 4.542 | 1.447 | 2.255 | 5.527 |
| *P* | 0.002 | 0.221 | 0.065 | <0.001 |
| Employment type |  |  |  |  |
| Bianzhi* | 18.63 ± 5.18 | 11.54 ± 2.98 | 18.55 ± 4.25 | 48.72 ± 8.44 |
| Contract-based employment | 17.32 ± 6.69 | 11.17 ± 3.00 | 18.66 ± 4.63 | 47.15 ± 11.31 |
| Others | 10.67 ± 7.23 | 10.00 ± 4.36 | 16.00 ± 4.00 | 36.67 ± 1.53 |
| *F* | 3.169 | 0.622 | 0.521 | 2.400 |
| *P* | 0.044 | 0.538 | 0.595 | 0.094 |
| Weekly working hours |  |  |  |  |
| ≤ 20 | 11.62 ± 6.48 | 9.62 ± 3.69 | 16.07 ± 6.21 | 37.36 ± 12.64 |
| 21-39 | 18.02 ± 5.87 | 11.50 ± 2.92 | 19.06 ± 4.05 | 48.58 ± 9.21 |
| 40-49 | 17.83 ± 6.41 | 11.32 ± 2.92 | 18.98 ± 4.28 | 48.14 ± 10.12 |
| ≥ 50 | 19.18 ± 5.01 | 11.60 ± 2.94 | 18.31 ± 4.31 | 49.09 ± 8.75 |
| *F* | 6.202 | 1.693 | 1.948 | 5.881 |
| *P* | <0.001 | 0.170 | 0.124 | <0.001 |
| Number of trials currently managed |  |  |  |  |
| 1-2 | 17.68 ± 5.91 | 11.18 ± 2.90 | 18.85 ± 4.23 | 47.70 ± 9.49 |
| 3-4 | 18.65 ± 5.77 | 11.05 ± 3.24 | 18.18 ± 4.54 | 47.88 ± 10.33 |
| 5-6 | 17.22 ± 6.60 | 12.52 ± 2.78 | 20.17 ± 3.69 | 49.91 ± 8.47 |
| > 6 | 16.56 ± 7.47 | 11.33 ± 2.85 | 16.50 ± 5.25 | 44.39 ± 13.24 |
| *F* | 0.693 | 1.451 | 2.636 | 1.024 |
| *P* | 0.558 | 0.230 | 0.051 | 0.383 |
| Systematic standardized training of clinical research |  |  |  |  |
| Yes | 16.86 ± 6.49 | 11.99 ± 3.18 | 18.27 ± 4.74 | 46.12 ± 10.51 |
| No | 19.89 ± 4.603 | 12.05 ± 2.44 | 19.21 ± 3.65 | 51.61 ± 8.13 |
| *t* | -3.566 | -2.441 | -1.453 | -3.489 |
| *P* | <0.001 | 0.016 | 0.149 | <0.001 |
| Employment status |  |  |  |  |
| Full-time job | 17.08 ± 6.36 | 11.64 ± 3.07 | 18.66 ± 4.10 | 47.38 ± 9.25 |
| Part-time job | 18.38 ± 5.88 | 11.09 ± 2.95 | 18.50 ± 4.69 | 47.96 ± 10.70 |
| *t* | -1.394 | 1.206 | 0.249 | -3.382 |
| *P* | 0.165 | 0.230 | 0.804 | 0.703 |

Note: *Bianzhi, a state-guaranteed permanent position with lifetime employment and comprehensive benefits; Data were presented as mean ± SD.

Table S3 Sensitivity analysis of job burnout total score using forced-entry multiple linear regression

| Factors | Unstandardised B (95% confidence intervals) | Unstandardised  SE | β | t | P value | VIF |
| --- | --- | --- | --- | --- | --- | --- |
| (Constant) | 43.269 (11.702, 74.836) | 15.986 | - | 2.707 | 0.008 | - |
| Gender | -2.453 (-10.640, 5.735) | 4.146 | -0.043 | -0.592 | 0.555 | 1.062 |
| Age | 0.333 (-0.532, 1.198) | 0.438 | 0.114 | 0.760 | 0.449 | 4.619 |
| Educational level | -2.048 (-5.080, 0.984) | 1.535 | -0.103 | -1.334 | 0.184 | 1.215 |
| Work experience | -0.469 (-3.578, 2.639) | 1.574 | -0.047 | -0.298 | 0.766 | 5.081 |
| Years as a CRN | -0.108 (-2.594, 2.377) | 1.259 | -0.009 | -0.086 | 0.932 | 2.100 |
| Professional title | -0.867 (-3.275, 1.541) | 1.220 | -0.069 | -0.711 | 0.478 | 1.931 |
| Employment type | -1.667 (-6.094, 2.760) | 2.242 | -0.064 | -0.744 | 0.458 | 1.505 |
| Weekly working hours | 0.332 (-1.994, 2.658) | 1.178 | 0.023 | 0.282 | 0.779 | 1.312 |
| Number of trials currently managed | -0.624 (-2.823, 1.574) | 1.113 | -0.044 | -0.561 | 0.576 | 1.279 |
| Systematic standardized training of clinical research | 2.160 (-2.226, 6.547) | 2.222 | 0.073 | 0.972 | 0.332 | 1.133 |
| Employment status | -1.367 (-5.355, 2.621) | 2.020 | -0.049 | -0.677 | 0.499 | 1.066 |
| Role ambiguity | 0.689 (0.306, 1.071) | 0.194 | 0.303 | 3.556 | < 0.001 | 1.483 |
| Role conflict | -0.427 (-1.204, 0.349) | 0.393 | -0.093 | -1.086 | 0.279 | 1.479 |
| Role overload | 0.681 (0.142, 1.221) | 0.273 | 0.218 | 2.493 | 0.014 | 1557 |

Note: VIF, Variance Inflation Factor
